# Quantitative chemical imaging of breast calcifications in association with neoplastic processes

**DOI:** 10.7150/thno.43325

**Published:** 2020-04-27

**Authors:** Kseniya S. Shin, Mint Laohajaratsang, Shuaiqian Men, Benjamin Figueroa, Suzanne M. Dintzis, Dan Fu

**Affiliations:** 1 Department of Chemistry, University of Washington, Seattle, 98195-1700, USA; 2 School of Medicine, University of Washington, Seattle, 98195-1700, USA; 3 Department of Pathology, University of Washington, Seattle, 98195-1700, USA

**Keywords:** Breast calcifications, breast cancer, chemical imaging, stimulated Raman scattering, second harmonic generation

## Abstract

Calcifications play an essential role in early breast cancer detection and diagnosis. However, information regarding the chemical composition of calcifications identified on mammography and histology is limited. Detailed spectroscopy reveals an association between the chemical composition of calcifications and breast cancer, warranting the development of novel analytical tools to better define calcification types. Previous investigations average calcification composition across broad tissue sections with no spatially resolved information or provide qualitative visualization, which prevents a robust linking of specific spatially resolved changes in calcification chemistry with the pathologic process.

**Method**: To visualize breast calcification chemical composition at high spatial resolution, we apply hyperspectral stimulated Raman scattering (SRS) microscopy to study breast calcifications associated with a spectrum of breast changes ranging from benign to neoplastic processes, including atypical ductal hyperplasia, ductal carcinoma in situ, and invasive ductal carcinoma. The carbonate content of individual breast calcifications is quantified using a simple ratiometric analysis.

**Results**: Our findings reveal that intra-sample calcification carbonate content is closely associated with local pathological processes. Single calcification analysis supports previous studies demonstrating decreasing average carbonate level with increasing malignant potential. Sensitivity and specificity reach >85% when carbonate content level is used as the single differentiator in separating benign from neoplastic processes. However, the average carbonate content is limiting when trying to separate specific diagnostic categories, such as fibroadenoma and invasive ductal carcinoma. Second harmonic generation (SHG) data can provide critical information to bridge this gap.

**Conclusion**: SRS, combined with SHG, can be a valuable tool in better understanding calcifications in carcinogenesis, diagnosis, and possible prognosis. This study not only reveals previously unknown large variations of breast microcalcifications in association with local malignancy but also corroborates the clinical value of linking microcalcification chemistry to breast malignancy. More importantly, it represents an important step in the development of a label-free imaging strategy for breast cancer diagnosis with tremendous potential to address major challenges in diagnostic discordance in pathology.

## Introduction

Calcifications associated with breast disease are critical to breast cancer screening as they are often the only discernible indicator of risk in mammography 1. Because of their importance in diagnostic radiology, it is essential to associate features of calcifications (e.g., distribution and morphology) with the ultimate pathology diagnosis. Several studies have found that casting-type calcifications are associated with carcinoma of high histopathological grade and more extensive disease 2-4. The assessment and identification of casting-type calcifications is based on architectural pattern, described with terms such as “intermittent” and “branching.” Carcinomas with casting-type calcifications are correlated with poor prognosis, including increased likelihood of lymph node metastasis, increased risk of recurrence, and decreased survival 5-7.

Despite their diagnostic and prognostic potential, the morphologic and chemical features of calcifications are poorly understood, in part due to technological limitations. Morphological features of calcifications identified by mammography are insufficient for accurate diagnosis and prognosis. In addition, smaller calcifications (<0.1mm) are undetectable using mammography 2. While histological studies can identify smaller (<0.1mm) calcification, they are mostly limited to crude morphological information and prone to sectioning artifact. Accumulating evidence demonstrating the importance of calcification type in breast cancer progression underscores the need to develop tools to more fully characterize calcifications 8-11.

Moving beyond morphology and distribution patterns in breast calcification, studies utilizing X-ray diffraction and electron microprobe analysis with scanning electron microscopy (SEM) have contributed to an earlier understanding of calcification chemical differences 11-14. The type I (calcium oxalate) calcifications were found in predominantly benign processes and often appeared translucent on hematoxylin and eosin (H&E) stained tissue slides. The type II (calcium hydroxyapatite) calcifications were found in both benign and malignant processes and appear intensely purple as they absorb hematoxylin on H&E slides. Spectroscopic methods such as Raman spectroscopy (RS) 15-19 and Fourier-transform infrared spectroscopy (FTIR) 20 demonstrated chemical differences in calcifications associated with benign and malignant breast disease. The carbonate content of hydroxyapatite has been shown to be inversely correlated with malignancy 20. Additional studies probed the correlation of malignancy with another component of microcalcifications - whitlockite (tricalcium phosphate and magnesium-substituted beta-tricalcium phosphate) 19,21-24.

While offering information about calcification chemistry, most spectroscopic methods average calcifications within multiple ducts within a tissue section, irrespective of the heterogeneous pathologic processes among adjacent ducts. Spatially resolved methods such as FTIR imaging have limited resolution and throughput, prohibiting visualization of calcification composition variation within adjacent ducts and lobules. Moreover, tissue protein content is recognized to correlate with malignancy 20,25, and small case set correlative studies provided supporting evidence of complex heterogeneity of calcifications 19,26. However, no detailed quantitative studies have been conducted focused exclusively on calcification underlying matrix co-localized with mineral components and diagnostic value of such information. To address this gap in understanding calcification variation within tissue microenvironments, we use stimulated Raman scattering (SRS) microscopy, an advanced Raman imaging technique, to map the chemical composition of breast calcifications (mineral constituents and underlying matrix) and associated neoplastic processes with unprecedented spatial resolution and chemical specificity. SRS uses two ultrashort laser pulses to coherently excite molecular vibrations with orders of magnitude higher efficiency than spontaneous Raman, thereby enabling rapid chemical imaging at submicron spatial resolution 27-29. The photon energy difference between the two lasers corresponds to the vibrational bond energy of the molecule of interest. By sequentially tuning the frequency difference of the two excitation lasers, different Raman modes can be selectively excited and allow construction of a hyperspectral SRS dataset with each spatial pixel containing an SRS spectrum. Based on the distinct Raman features of target molecules, they can be separately imaged and quantified. SRS has now been widely used to quantify the spatial distribution of various soluble small molecules such as neurotransmitters, metabolites, and small drug molecules 30-32. SRS has also shown promise for label-free histopathology applications, where the intrinsic vibrational contrasts of lipids and proteins are used to provide H&E equivalent information without any sectioning, fixation, or staining 29,33-35.

We utilize the high resolution and chemical specificity SRS offers to deliver an unprecedented level of detail when analyzing calcifications. Additionally, we incorporate second harmonic generation (SHG) which has been proven effective at visualizing collagen in the tumor microenvironment 36. SHG added to SRS helps visualize collagen, including both stromal collagen and collagen within the calcification matrix. Our results reveal significant intra-patient heterogeneity of calcifications that are tightly associated with the underlying pathological process. Interpatient analysis demonstrates that the carbonate content percentage decreases as the malignancy potential increases. Moreover, calcifications have protein-containing underlying matrix that is unique to specific neoplastic processes. Finally, we determine that carbonate content, in combination with SHG data, is very effective in separating benign from neoplastic processes.

## Experimental Section

### SRS and SHG imaging

**Figure 1:**
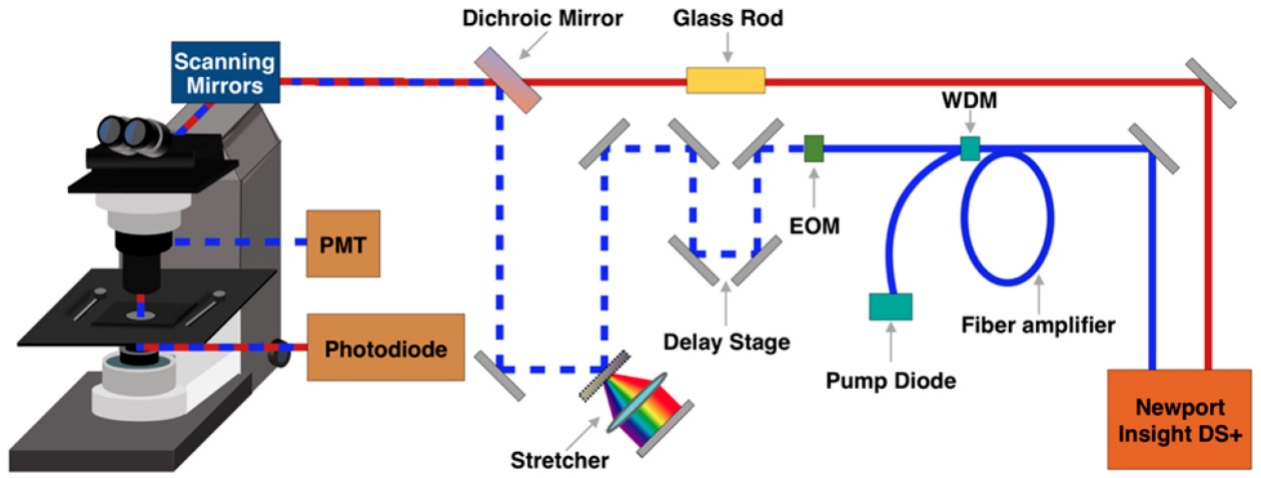
Schematic representation of stimulated Raman scattering (SRS) and second harmonic generation (SHG) microscopy experimental setup. EOM - electro-optic modulator. PMT - photomultiplier tube. WDM - wavelength division multiplexer.

A broadband femtosecond dual-beam laser system (Insight DS + from Spectra-Physics) was used for SRS and SHG (Figure 1). The details of the experimental setup for broadband SRS measurements has been described in a previous publication 37. Briefly, the two output beams from the Insight laser system are used at 80 MHz repetition rate: a tunable beam (pump) and a fixed beam (Stokes). The pump beam is centered at 798 nm for CH region and 944 nm for fingerprint region. The Stokes beam is amplified with a parabolic fiber amplifier to increase the bandwidth and pulse duration 37. We note that the use of this laser is critically important for this study because of the improved spectral resolution, coverage, and background suppression. Temporally dispersed pump and Stokes pulses are combined at a dichroic mirror and sent into a home-built laser scanning microscope. A 25x Olympus water immersion objective (NA = 1.05) is used to focus the beams on the sample. At the focus, the pump and Stokes beams have an average power of 40 mW each. Stimulated Raman loss (SRL) signal is detected with an amplified photodiode and a lock-in amplifier (Zurich Instrument HF2LI). For SRS imaging, a stack of frames with single field of view (FOV) of 285 μm x 285 μm (512 pixels x 512 pixels) is acquired, for which the pixel dwell time is 8 μs and time to acquire each frame is 2 s. The frames are acquired every 2 cm^-1^ for total of 90 frames per stack. For SHG, a dichroic mirror (Chroma 695dcxr) is placed before the objective to allow for epi-collection of SHG signal from the sample. The SHG signal is detected by a photo-multiplier tube (Hamamatsu H10770PA-40) using 485 nm long pass and 650 nm short pass filters.

### Calibration sample preparation

Calcium hydroxyapatite (HAP) and 10% carbonated hydroxyapatite (CHAP) were obtained from Sigma Aldrich and Clarkson Chromatography Products respectively. Both solid controls were ground using mortar and pestle. A total of five mixtures were prepared for calibration purposes (0%, 2.5%, 5.0%, 7.5%, 10% carbonate content). The calibration samples were placed between a coverslip and a microscope slide for SRS imaging.

### Breast tissue specimens

Breast tissue was obtained from 17 patient archival cases (including biopsy and resection) managed by Northwest Biotrust, Seattle, WA. The initial case selection was based on the pathology report (calcifications present in association with desired pathological process). Slides were subsequently reviewed to confirm the histologic diagnoses (SMD, KSS). Formalin-fixed paraffin-embedded tissue blocks were retrieved from archives and sectioned at 4-microns and mounted on charged slides. One section was H&E stained to identify pathological process. The adjacent section was deparaffinized and coverslipped for SRS and SHG analysis. The areas of interest were identified in the bright field based on selected areas of interest on adjacent H&E stained slides. A total of 214 breast calcifications were imaged, including: 31 non-neoplastic calcifications (including normal ducts and adenosis), 27 fibroadenoma (FA) associated calcifications, 8 atypical ductal hyperplasia (ADH) associated calcifications, 36 ductal carcinoma in situ (DCIS) associated calcifications, 112 invasive ductal carcinoma (IDC) associated calcifications. Supplementary Table 1 contains more detailed case description and additional clinical history.

### Quantification of carbonate content in hydroxyapatite

**Figure 2:**
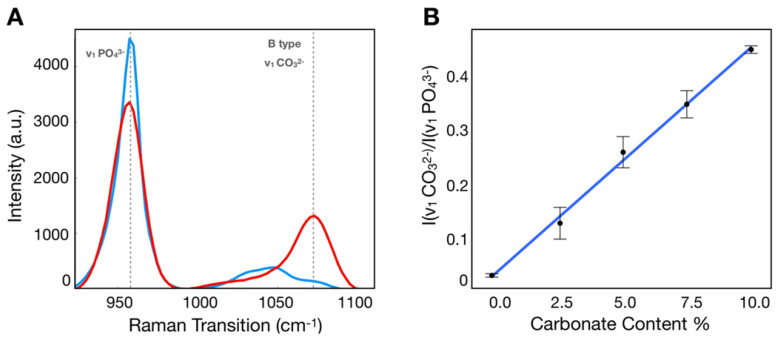
Calibration process to determine carbonate content in hydroxyapatite. A) SRS spectra of hydroxyapatite (blue) and carbonated hydroxyapatite (red) controls. B) Calibration curve correlating ratio of peaks at carbonate and phosphate Raman transitions.

Calibration samples with 0%, 2.5%, 5.0%, 7.5%, 10% carbonate content in calcium hydroxyapatite mixtures were imaged using the hyperspectral SRS microscope described above. Using SRS intensities at carbonate (~1070 cm^-1^) and phosphate (~960 cm^-1^) peaks corrected for background 38, the ratio was determined for each mixture (Figure 2A), which was plotted as a function of carbonate content percentage (Figure 2B). Using linear regression, the slope and intercept were determined and applied to convert the ratio described above into carbonate content percentage in hydroxyapatite.

### Tissue imaging and image processing

**Figure 3:**
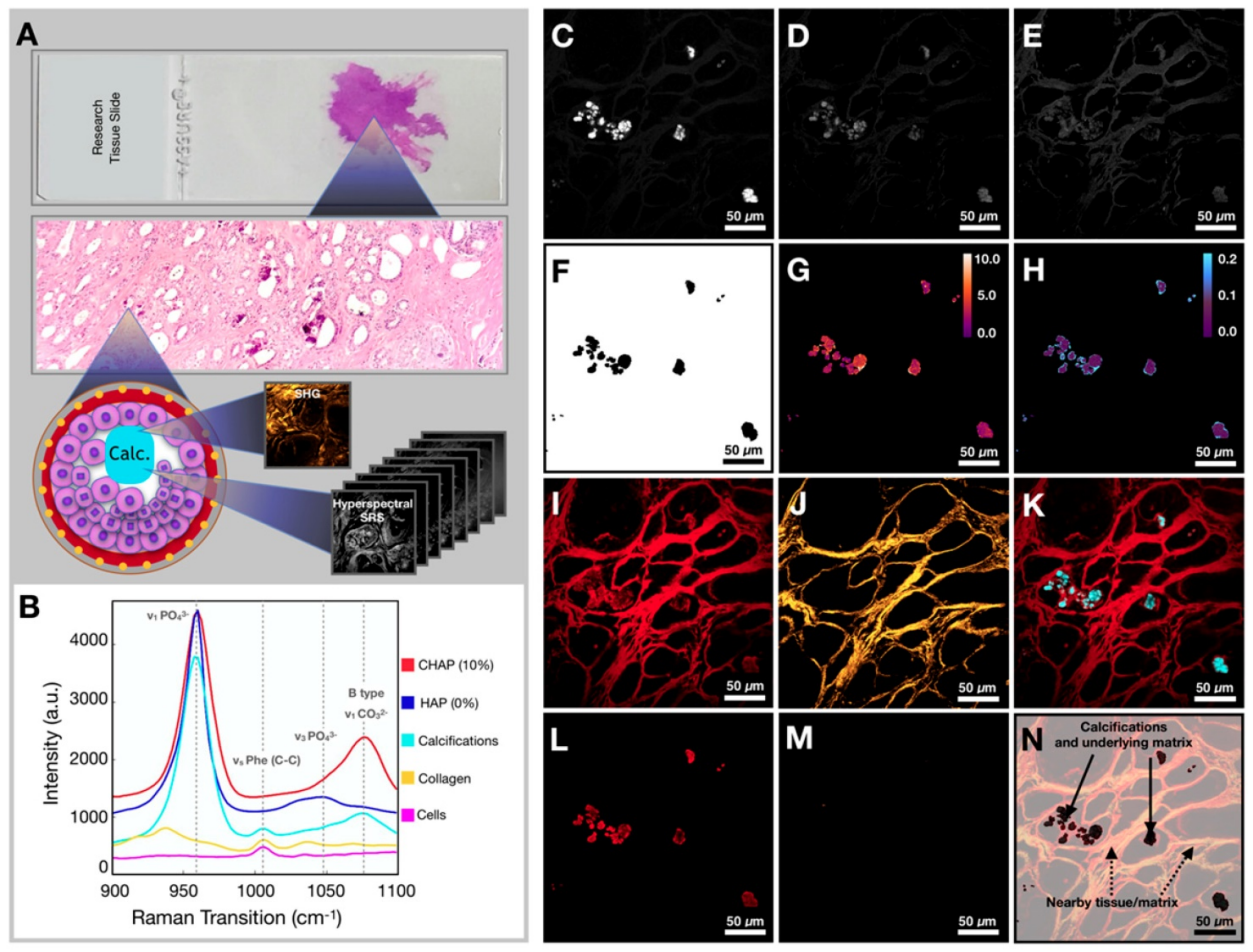
Workflow for tissue imaging and image processing. A) Diagram depicting imaging of the field of view to yield hyperspectral SRS and SHG information. B) Spectra of controls (CHAP and HAP) and spectra from various components encountered in breast tissue (calcifications, collagen, and ductal cells). C) Image at phosphate transition (~960 cm^-1^). D) Image at carbonate Raman transition (~1070 cm^-1^). E) Image at phenylalanine Raman transition (~1005 cm^-1^). F) Phosphate image-based mask used to isolate calcifications only. G) Carbonate content % map (mask applied). H) Phenylalanine to phosphate ratio map (mask applied). I) Image at CH Raman transition (~2930 cm^-1^). J) SHG image. K) Composite of CH (red) and phosphate (cyan) images. L) CH Image (mask applied). M) SHG image (mask applied). N) Diagram depicting nomenclature used in the manuscript.

Our imaging and data processing workflow are described in Figure 3A. Samples were imaged in both high wavenumber (C-H stretching) and fingerprint region of the Raman spectrum. The hyperspectral datasets were acquired in both regions. Figure 3B shows the SRS spectra of controls (CHAP and HAP) used to verify identified calcifications as well as collagen and cellular material (see Supplementary Figure 1 for a focused presentation of cell bodies that are not easily visualized in current figure). SRS images at phosphate and carbonate Raman transitions (Figures 3C and 3D) were background corrected and divided to generate a map of carbonate to phosphate ratio. The mask based on phosphate intensity (Figure 3F) was applied to the images to isolate only data co-localized with phosphate (the main calcification component). Using the calibration curve determined from control samples, the ratio value was converted to carbonate content percentage (Figure 3G). Images at phosphate and phenylalanine Raman transitions (Figures 3C and 3E) were used to generate a phenylalanine to phosphate ratio map (Figure 3H). Supplementary Figure 2 depicts additional examples of image processing. Additionally, the SRS image corresponding to a protein Raman transition (~2930 cm^-1^) was used to visualize tissue morphology of the FOV (Figure 3I). The SHG intensity image from the same FOV was collected to provide information on collagen (Figure 3J). The combination of protein and phosphate images provided visualization of the calcification location (Figure 3K). The mask (Figure 3F) was applied to CH (Figure 3L) and SHG images (Figure 3M). While the CH image was only used for verification of calcification underlying matrix (Figure 3N), the SHG image was masked using the same phosphate-based mask to isolate SHG signal only from collagen within calcifications for further statistical analysis.

### Statistical analysis

For each parameter (carbonate content percentage, phenylalanine to phosphate ratio, and SHG intensity) the average, for a given category (benign, ADH, DCIS, IDC, FA), was calculated with the standard deviation reported as an error bar. The differences between categories were assessed using p-values calculated from Student's t-tests. The receiver operator curves (ROC) were calculated using R software to assess the sensitivity and specificity for each parameter. Maximum Youden's index was used to determine sensitivity and specificity values.

## Results and Discussion

### SRS imaging provides spatially resolved quantification of carbonate content in hydroxyapatite

Previously, the carbonate content in hydroxyapatite has been measured using RS 16,18,25,39 without detailed understanding of spatial distribution. Here, we use pure HAP and commercially synthesized 10% CHAP to demonstrate that ratiometric analysis of SRS images can deliver a high-resolution spatial map to assess variations of carbonate content in CHAP. Reproducible and reliable quantification of carbonate content is necessary to study how tissue microenvironment influences calcification characteristics.

**Figure 4:**
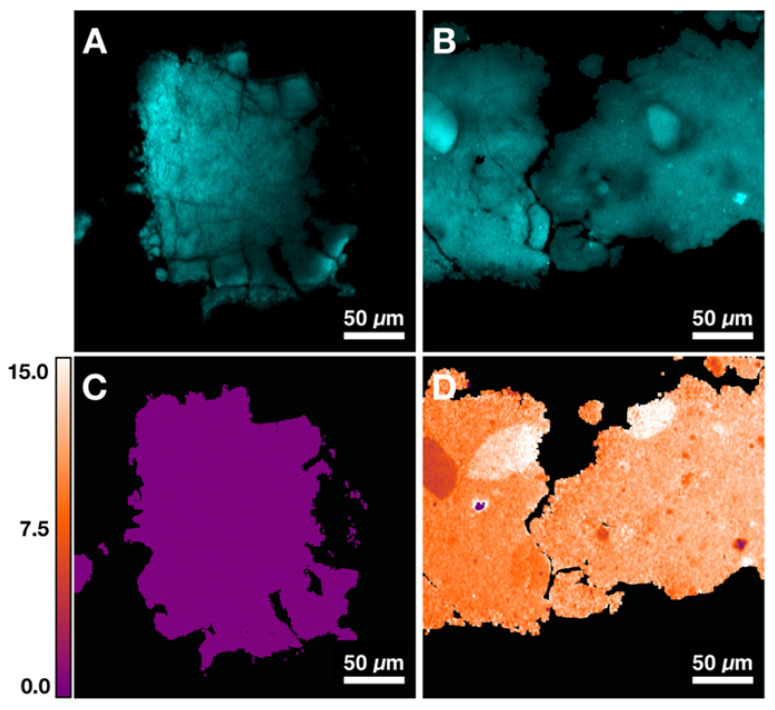
Quantification of carbonate content in hydroxyapatite (HAP) based on control samples. A) Image at phosphate Raman transition (960 cm^-1^) for pure HAP. B) Image at phosphate Raman transition (960 cm^-1^) for 10% carbonated hydroxyapatite (CHAP). C) Carbonate content map for HAP. D) Carbonate content map for 10% CHAP.

Figures 4A and 4B demonstrate two control samples (HAP and 10% CHAP) imaged at phosphate Raman transition. Applying calibration to ratiometric SRS image of carbonate/phosphate, the carbonate content percentage maps are generated for both control samples. Figure 4C shows HAP as an image with almost uniform carbonate % of 0.00 (±0.00) as expected with pure HAP. Figure 4D shows heterogeneous CHAP with average carbonation substitution % of 10.34 (±1.87) due to the nonuniformity of the synthetically prepared sample. Using the high resolution and chemical specificity of SRS, we are able to resolve the fine fluctuations of carbonate content with submicron resolution. This unique capability allows us to assess the effect of metabolic changes precipitated by presence of cancer in breast tissue on calcification composition.

### Calcifications are heterogenous within a single patient

Breast tissue submitted for pathological examination often contains calcifications in multiple ducts, which may represent various pathologic processes. In previous spectroscopic studies, multiple ducts are frequently measured together, which can average calcifications associated with different processes. Isolating individual breast ducts and detailing the histopathology within the ducts allows for correlation of variation in carbonate content with local pathology.

**Figure 5:**
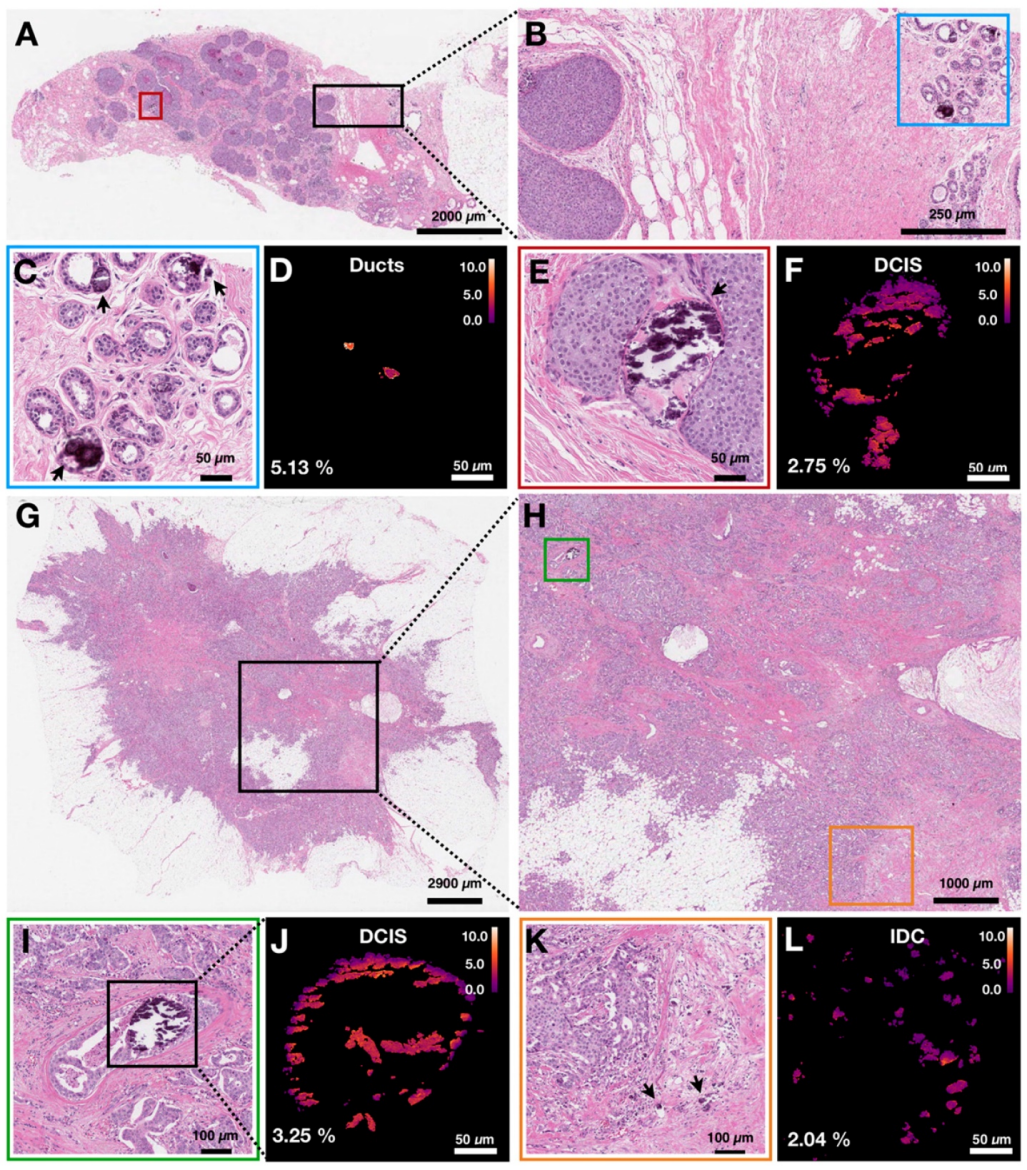
Carbonate content in calcifications varies within individual patient samples. Arrows are used to identify calcifications. A) H&E of breast biopsy core with red box highlighting DCIS ducts and black box highlighting normal ducts in close proximity to DCIS lesion edge. B) H&E close up of area highlighted in black box in A. Blue box highlights normal ducts. C, D) H&E close up and carbonate content map, respectively, of normal ducts highlighted in B. Arrows point to calcifications. E, F) H&E close up and carbonate content map, respectively, of ducts containing DCIS with associated calcifications highlighted in A. G) H&E of breast tissue resection with black box highlighting an area of DCIS admixed with IDC. H) H&E close up of area highlighted in black box (see G). Green box highlights a duct containing DCIS with associated calcifications. Orange box highlights IDC with associated calcifications. I, J) H&E close up and carbonate content map, respectively, of ducts containing DCIS with associated calcifications highlighted in H. K, L) H&E close up and carbonate content map, respectively, of IDC with associated calcifications highlighted in H.

Here, we group calcifications by specific pathological processes and not by overall case diagnosis. In Figure 5, we demonstrate two cases in which multiple pathological processes co-occur within the same tissue slide. Figure 5A shows an H&E stained section of breast biopsy core with normal ducts and a focus of DCIS. Figure 5B highlights the proximity of the normal ducts to the edge of DCIS focus. The H&E close up of normal ducts reveals multiple calcifications (Figure 5C). The corresponding carbonate content map in Figure 5D shows the carbonate content average of 5.13%. Calcifications from the DCIS focus are shown in Figure 5E. The corresponding carbonate content map in Figure 5F shows an average carbonate content of 2.75%.

Figure 5G shows H&E stained section of another breast resection tissue with calcifications associated with DCIS as well as IDC. Figure 5H highlights the proximity of the DCIS containing ducts to the calcifications associated with IDC. The stroma surrounding a duct containing DCIS is infiltrated by carcinoma cells. Figures 5I and 5J show the close-up H&E and a carbonate content map for calcifications associated with DCIS, respectively. The average carbonate content is 3.25%. Figures 5K and 5L show the close-up H&E and a carbonate content map for calcifications associated with invasive component, respectively. The average carbonate content value is 2.04%.

The two cases illustrate that calcifications in the same patient case can have differing chemical composition. Although ducts are present in the same tissue section, the carbonate content associated with individual ducts is markedly different and reflects underlying pathological process. The high spatial resolution of hyperspectral SRS imaging is important to resolve calcification heterogeneity within the same patient sample and to establish the relationship between chemical composition and pathological process.

### SRS imaging provides visualization of local breast tissue microenvironment and its influence on calcifications

Previous studies using FTIR have demonstrated that breast calcifications with lower carbonate content of CHAP correlate with malignancy 20. Moreover, the overall protein content in tissue was noted to increase with malignancy 20,25. Breast calcifications may contain both organic material and mineral components. The relative amount of organic material is likely to vary in normal compared to neoplastic ducts. Furthermore, the extracellular pH becomes acidic in neoplastic tissue as a result of lactate secretion from anaerobic glycolysis 40,41 and lower pH is likely to contribute to lower carbonate content in malignant calcifications. We hypothesize that detailed visualization of carbonate content and organic components in calcifications will reveal important features that can aid breast cancer diagnosis.

**Figure 6:**
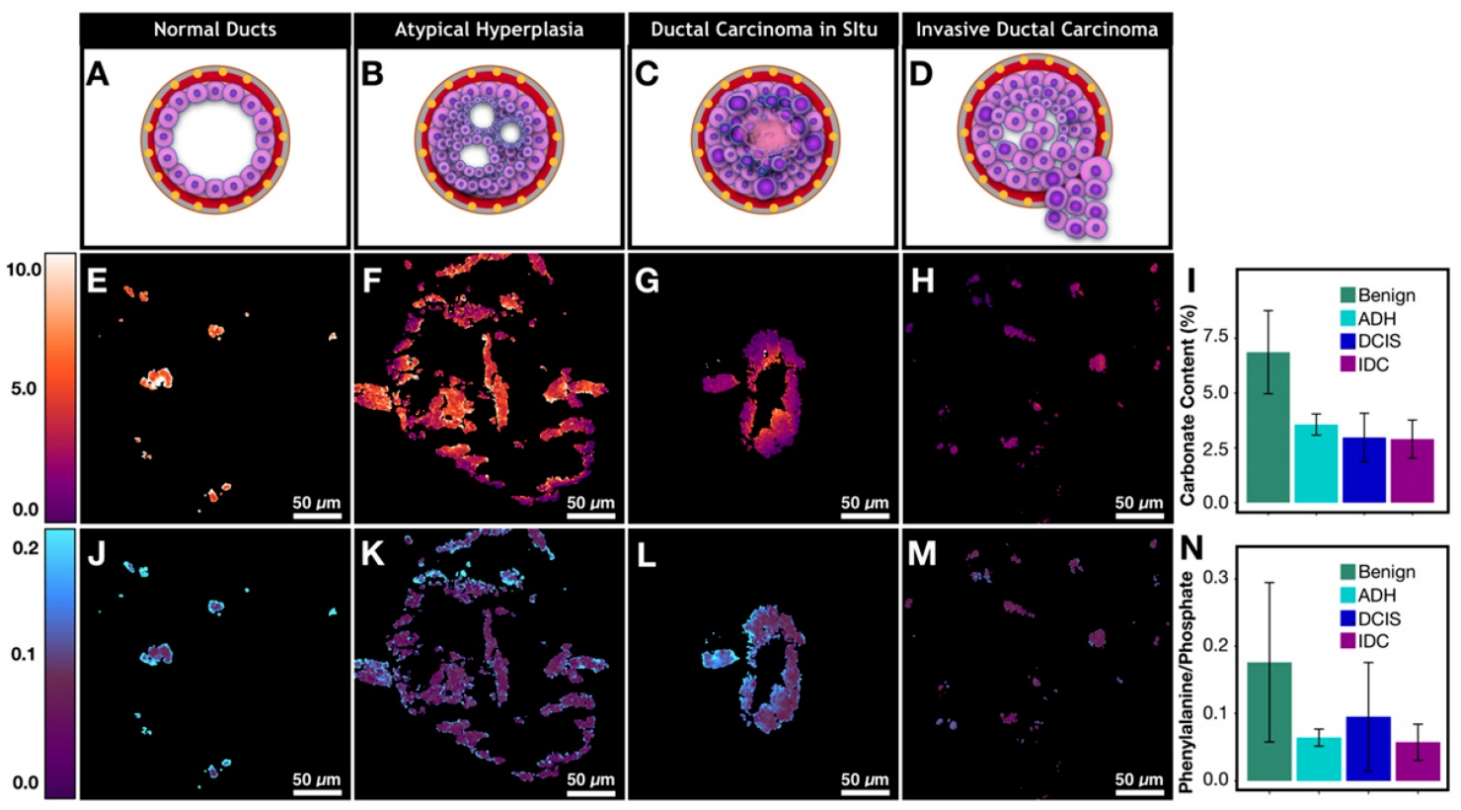
Metabolic changes associated with cancer affects calcification composition. A)-D) Graphic depiction of neoplastic progression from normal duct to invasive ductal carcinoma. E)-H) Carbonate content changes with neoplastic progression. I) Bar graph demonstrating averages for carbonate content across pathological categories. J)-M) Phenylalanine to phosphate ratio changes with neoplastic progression. N) Bar graph demonstrating averages for phenylalanine to phosphate ratio across pathological categories.

Figures 6A-D demonstrates a graphic depiction of normal ducts progressing to IDC. The cells of the ducts undergo a series of mutations that allow bypassing of the normal checks and balances of the cell cycle. Once a cell is able to divide without internal or external controls, clonal expansion ensues. Invasive carcinoma acquires the ability to break through the basement membrane surrounding ducts and invade the stroma (gray border as depicted in Figures 6A-D), thereby conferring risk for lymphovascular invasion and subsequent metastasis to other organs.

The neoplastic progression from benign ducts to IDC is associated with an overall decrease in calcification carbonate content (Figure 6I, Supplementary Table 2 for p-values). Our results support previous findings from spectroscopic studies 20. Decreased carbonate content is correlated with malignancy and by extension with increased cellular metabolism and uncontrolled division. Specifically, normal ducts have high carbonate content that averages around 6.9%. Meanwhile, atypical hyperplasia and DCIS calcifications (Figures 6F and 6G) show low carbonate content on the edges with values of 1-2% while the carbonate content in the center of ducts (100-300 μm in diameter) is much higher, approaching the values associated with non-neoplastic processes. Interestingly, when average carbonate content is grouped on a per patient basis, the difference between benign and malignant cases is much less pronounced (supplementary Figure 3). This is due to the fact that local pathology around some calcifications may not agree with overall patient diagnosis. The discrepancy further highlights the advantage of calcification imaging over spectroscopy of bulk tissue and the need for spatially resolved measurement in heterogeneous tumor tissue, particularly when tissue size is limited such as in needle core biopsy.

Although precise quantification of the spatial distribution of carbonate in large calcifications is challenging due to sectioning artifact, our findings suggest that the carbonate content decrease on the edges of the calcifications directly reflects the changing microenvironment (i.e. higher acidity) associated with neoplastic cells and necrotic debris 40,41. With invasive disease, the calcification carbonate content is very low (1-3%) and there is lower variation across a single calcification (Figure 6H). Together these observations suggest that low carbonate content on the edge of calcifications could potentially be used as a neoplastic indicator. A broader validation of this finding would benefit from unsectioned tissues samples where calcifications are not perturbed by microtome cutting.

In addition to carbonate content of hydroxyapatite, protein to phosphate ratio has also been suggested to correlate with breast cancer 20. We are interested in understanding how calcifications interact with underlying tissue matrix. Using phenylalanine as a surrogate marker for protein 42,43, phenylalanine (~1005 cm^-1^) to phosphate (~960 cm^-1^) ratio is used to quantify protein content in calcifications (Figures 6J-M). The phenylalanine to phosphate ratio decreases overall when comparing benign breast to invasive carcinoma (Figure 6N, Supplementary Table 2 for p-values). Additionally, there is a notable increase of protein content in DCIS. The ratio of the phenylalanine increases especially on the edges of the calcifications. The differences can be explained by necrosis that is present in many DCIS cases (a representative case is shown in Figure 6L) providing a lot of organic material to be admixed with calcifications.

Many theories have been put forth to explain different processes that can lead to calcifications associated with a specific pathological process. In necrotic tissue, it is believed that the areas of cell necrosis or previous microscopic cell injury activate phosphatases that bind calcium ions to phospholipid in the membrane, which incites further calcification 44. This explains why calcifications formed in necrotic tissue are likely to be mixed with the necrotic debris and more heterogeneous, as supported by our observations of higher phenylalanine to phosphate ratio and larger variations in DCIS with necrosis (Figure 6L).

Moreover, recent studies have described an epithelial-mesenchymal transition of neoplastic cells that become capable of producing breast calcifications in breast carcinoma 45. As transitioned cancerous cells invade stroma, calcifications are likely to be produced frequently and are likely to be relatively homogeneous with low organic content in the absence of loose organic material in the stroma. This mechanism is supported by our findings that show invasive cancer foci with small scattered calcifications and with relatively homogenous and low organic content (Figure 6M).

Overall, the SRS images reveal chemical features associated with the calcification microenvironment. Carbonate content is decreased at the edges of DCIS calcifications. Patterns such as these can only be highlighted with high resolution and chemical specificity available with hyperspectral SRS. Moreover, ratiometric phenylalanine to phosphate imaging can quantitate protein within calcifications. We observed a significant increase of protein content in DCIS compared to IDC. With additional validation, the calcification features imaged with SRS could become a part of an invaluable diagnostic tool.

### Calcification and underlying protein matrix contain diagnostically relevant information

While phenylalanine SRS image offers an overall measure of protein content, it cannot provide details regarding the structure of organic material within the calcifications. Radiographic studies of calcification morphology and texture show that calcifications differ in various pathological processes 4,46. The underlying organic matrix of calcifications, determined by the cellular process, affects mineralization. The composition of the underlying organic matrix varies considerably between FA and IDC. In past spectroscopic studies 20, fibroadenomas were grouped with non-neoplastic cases. FA is in fact a result of neoplastic stromal component and is hormonally responsive 47. Using SRS, we found that FA has low carbonate content in calcifications. Low carbonate content by itself might lead to misclassification as malignant.

**Figure 7:**
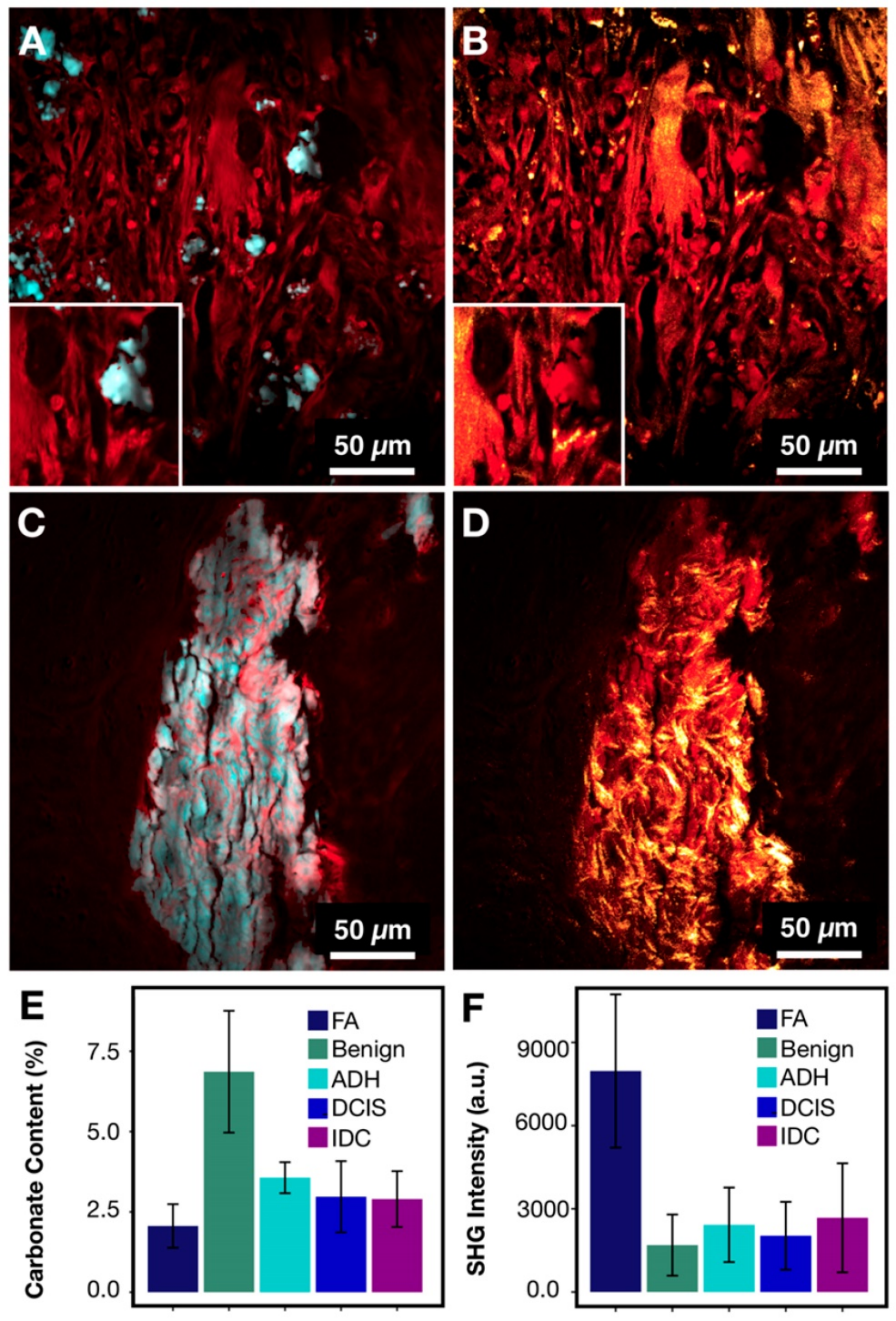
FA and IDC similarity and differences. A) IDC with protein (red) highlighting calcification underlying matrix along with stroma and phosphate (cyan) highlighting hydroxyapatite. B) IDC composite of protein (red) and SHG (gold) highlighting collagen. C) FA with protein (red) highlighting calcification underlying matrix along with stroma and phosphate (cyan) highlighting hydroxyapatite. D) FA composite of protein (red) and SHG (gold) highlighting collagen. E) Bar chart for carbonate content across all categories. F) Bar chart for SHG intensity across all categories.

Figures 7A and 7C present protein information combined with phosphate as an example of common morphology for IDC and FA associated calcifications, respectively. From the images, the FA cases frequently have large and very dense calcifications, whereas IDC cases tend to present with smaller scattered calcifications. These basic morphological findings are consistent with mammography studies. Often on mammography, FA associated calcifications are described as popcorn calcifications. Such calcifications can appear dense, thick, and usually large (~2 mm) 48,49. In contrast, IDC is often associated with crushed stone, powdery, or casting-type calcifications 46,50. While the morphological differences are present for FA cases when compared to IDC cases, the carbonate content of hydroxyapatite is less differentiating with average of 2.24% *versus* 3.01% for FA and IDC respectively.

On mammography, FA calcifications can persist over many years, whereas cancer associated calcifications can appear on mammography within months 48. This temporal difference in calcification formation suggests that the underlying matrix organization may be a useful differentiation factor. Figures 7B and 7C show protein information (red) combined with SHG (gold). In FA cases, the SHG signal is prominent and highlights a basket weave pattern. In contrast, in IDC cases, the SHG signal highlights mostly stroma, while the calcifications themselves do not give strong SHG. Figures 7E-F summarize the averages of SHG signals for different pathological categories. The average SHG signal for FA is almost three time that of IDC.

In addition to distinguishing between FA and IDC, our findings offer a plausible explanation of why FA calcifications appear to be dense, large, and popcorn like. The denser organic matrix and intricate collagen network likely supports and stabilizes larger calcifications associated with FA that frequently remain unchanged for decades. In contrast, the absence of collagen or structure-reinforcing matrix in IDC calcifications lacks the structural integrity and often appear small and powdery.

In summary, FA cases can be differentiated from malignant cases using a combination of data from SRS and SHG. The differentiation is clinically crucial due to substantially different management of FA vs IDC cases. Moreover, the visualization of FA calcification underlying matrix correlates with previous radiological data.

### Combined SRS/SHG imaging aids differentiation of neoplastic cases

Distinguishing IDC from benign ducts using standard histology is typically not considered challenging 51. However, cases with ambiguous non-neoplastic *versus* neoplastic morphology, such as atypical ductal hyperplasia, demonstrate low interpathologist agreement 51. Combining traditional H&E morphology with specific chemical signatures of calcifications could help with the overall interpretation of borderline cases and subsequently improve the diagnostic accuracy and patient outcome.

**Figure 8:**
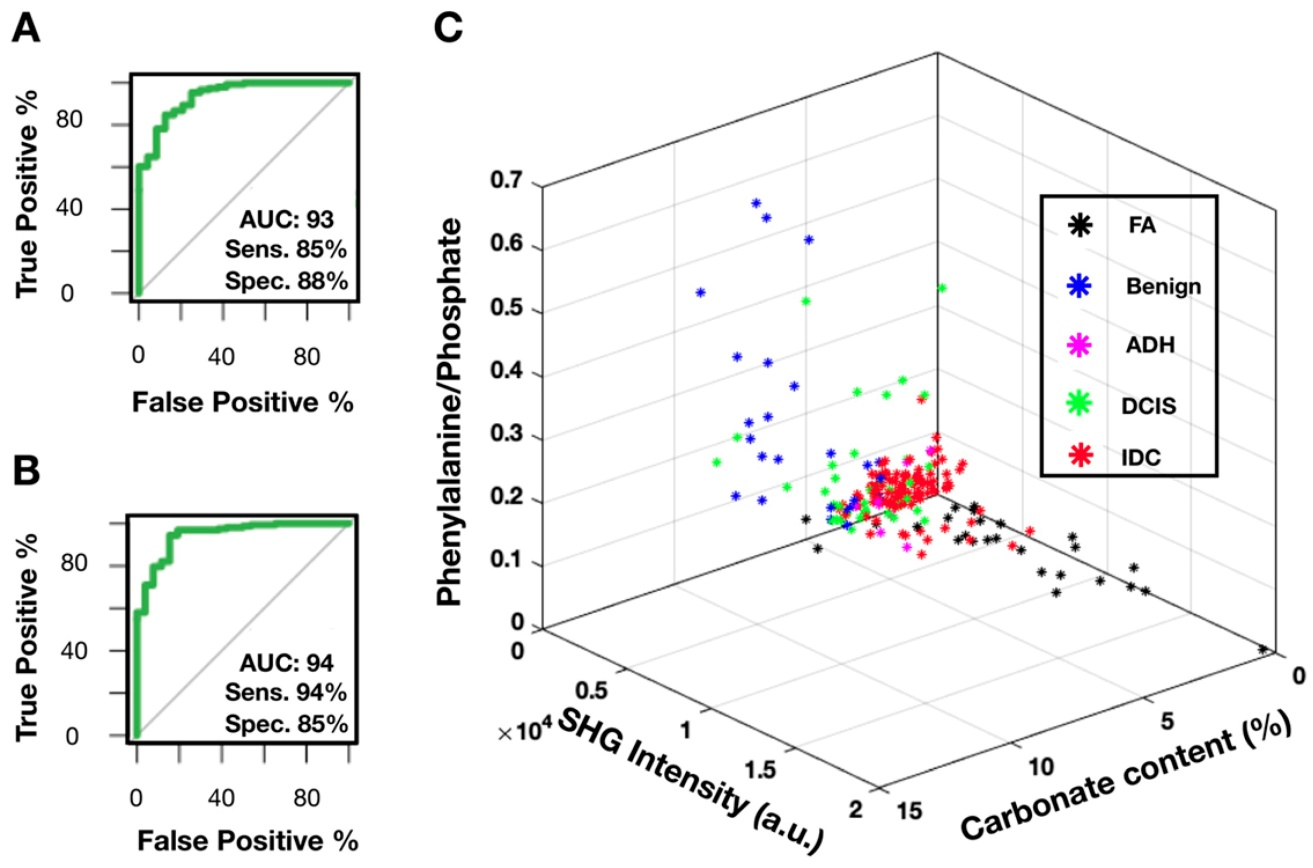
SRS and SHG imaging data statistical analysis. A) ROC curve for carbonate content as a parameter when differentiating benign and neoplastic cases. B) ROC curve for SHG intensity as a parameter when differentiating FA and IDC cases. C) Scatter plot depicting separation of cases across all diagnostic categories.

Figure 8A shows the receiver operating characteristic (ROC) curve for breast cancer diagnosis using carbonate content alone. The area under the curve (AUC) of 0.93 (1 for perfect model) suggests that carbonate content is a sufficient metric to separate benign from neoplastic cases. The AUC of 0.8 to 0.9 is considered excellent and above 0.9 is considered outstanding in medical practice 52. In comparison, most recent published research 24 presented a complex classification model using RS data and reported ROC of 0.94 (when differentiating only pure benign and pure malignant cases). Moreover, the sensitivity of 85% and specificity of 88% as determined by maximum Youden's index is comparable to other studies utilizing carbonate content in conjunction with other parameters such as protein 20,25. In our case, the carbonate content alone appears to be very effective.

Provided our average carbonate content for FA category is similar to IDC, another parameter, SHG, is utilized to separate these diagnoses. Figure 8B presents a ROC curve for FA *versus* IDC determination based on SHG signal alone. The AUC of 0.94 with sensitivity of 94% and specificity of 85% as determined by maximum Youden's index supports previous observations that FA cases have markedly increased SHG signal. In contrast, IDC cases were observed to have minimal SHG signal. Using SHG intensity as a parameter, we can separate FA cases, a benign neoplastic process, from cancer.

Combining SRS determined carbonate content, phenylalanine to phosphate ratio, and SHG data, we demonstrate a successful separation for all diagnostic categories (Figure 8C). Benign calcifications are grouped using carbonate content with low average SHG intensity. Interestingly, DCIS (highlighted in green) calcifications lie somewhere between benign and invasive, the majority closer to invasive. Additionally, ADH mostly clustered adjacent to DCIS. This co-localization of ADH and DCIS is consistent with the general understanding that ADH is not a unique biological category, but rather a diagnostic catch-all designated to indicate ambiguous morphology. The consensus amongst pathologists is that many ADH cases are biologically indistinguishable from low-grade DCIS.

In summary, SRS data provides high sensitivity (85%) and specificity (88%) when identifying benign and invasive processes. SHG data provides a parameter separating FA from IDC cases with high sensitivity (94%) and specificity (85%). Phenylalanine to phosphate ratio provides additional separation between DCIS and IDC. Together, spectroscopy and morphology can be utilized to better establish the malignant potential in cases with ambiguous morphology on H&E alone.

### Additional mineral species and their effect on ratiometric analysis

CHAP is not the only microcalcification species that was identified in breast cancer. Although whitlockite typically accounts for a tiny percentage of breast calcifications 22, it has been suggested that the presence of whitlockite species (tricalcium phosphate and magnesium substituted tricalcium phosphate) could potentially aid diagnosis 19,21-24. However, the experiments to date have yielded conflicting results with some studies linking whitlockite to malignancy 19,21,22, and others associating whitlockite more frequently with benign processes 24.

**Figure 9:**
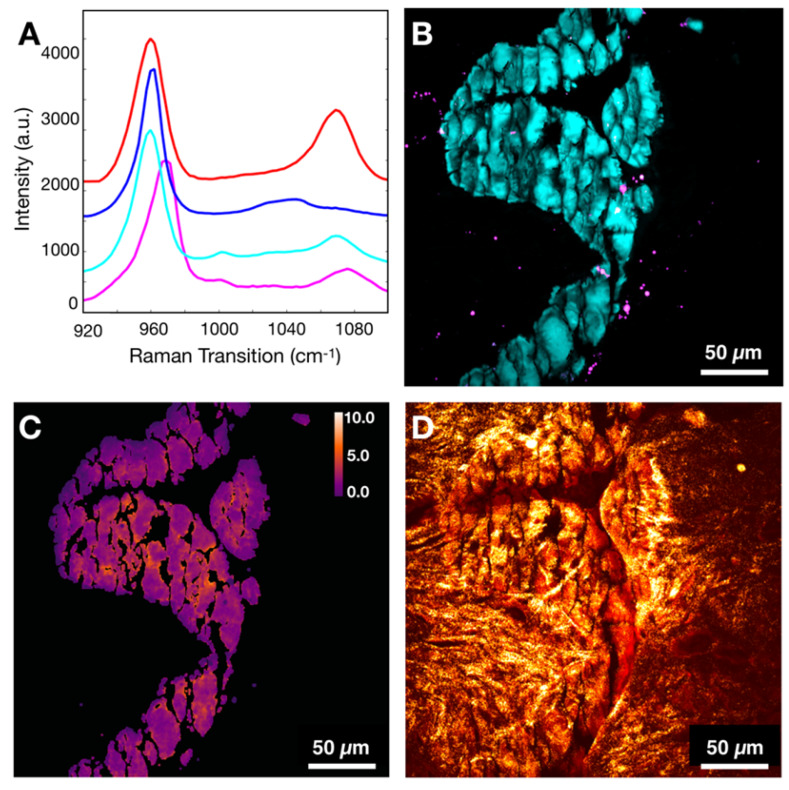
Evaluation of whitlockite contribution to dominant mineral species HAP/CHAP. A) Spectral differences of CHAP (red), HAP (blue), FA associated CHAP/HAP (cyan), and whitlockite (magenta). B) Depiction of spatial distribution of HAP/CHAP highlighted using phosphate (cyan) and whitlockite highlighted using red-shifted phosphate (magenta). C) Carbonate content map for the same FOV. D) Calcification underlying organic matrix and stroma depicted using CH Raman transition (red), and SHG (gold).

Typically, whitlockite can be identified using Raman spectroscopy due to unique spectral features (red-shifted phosphate peak from 960 cm^-1^ to 970 cm^-1^) 53. Our hyperspectral data sets were screened for additional mineral species and whitlockite was identified only in fibroadenoma cases. Figure 9A demonstrates representative spectra for CHAP, HAP, FA associated calcifications composed of CHAP/HAP, and whitlockite. The spectrum of whitlockite demonstrates a red-shift as shown before 53. Figure 9B shows a spatial distribution of whitlockite relative to dominant species of CHAP/HAP in a representative FA case. As expected, whitlockite is not a prominent species, which is in agreement with previously published estimates of 2% or less for whitlockite identified in calcifications 22.

Provided the spectral differences in CHAP/HAP and whitlockite, it is expected that the calculated carbonate content % for pixels attributed to whitlockite is likely to introduce a slight error if not excluded. Without considering the presence of whitlockite, the average carbonate content is 2.35% for Figure 9C. When considering the presence of whitlockite (by removing any pixels contributed by whitlockite), the average carbonate content is 2.38%. This suggests an error of less than 2% if we fail to include whitlockite. For most other cases, this error is even less due to lower content of whitlockite.

Figure 9D shows CH image in combination with SHG data to demonstrate collagen presence in both stroma and calcifications themselves. Although it is not clear why different studies disagree on what cases have whitlockite frequently associated with, it is possible that the underlying matrix does matter when considering what fragments of calcifications make it through tissue processing, paraffin embedding, and microtome cutting. To fully determine the contribution of whitlockite in microcalcifications and its diagnostic utility, imaging of fresh unprocessed tissue is needed.

## Conclusion and Future Outlook

Breast calcifications are used to guide patient biopsy prior to resection. However, morphologic assessment of calcifications in mammograms provides limited diagnostic information. Spectroscopic techniques such as spontaneous RS have shown promise for in vivo breast cancer screening in conjunction with mammography. However, to date, detailed spatial and microenvironmental distribution of calcifications in carcinogenesis is poorly understood. Here, we report the first quantitative high-resolution SRS imaging of calcifications, and their association with nearby tissue matrix as well as visualization of calcified organic matrix components with SHG. We developed a simple method to quantify the carbonate content of hydroxyapatite, the main calcification species associated with cancer, based on the ratio of carbonate to phosphate Raman peak. Applying this method to a diverse set of breast calcifications associated with a range of neoplastic processes, we revealed that the microenvironment of the neoplastic processes strongly influences the local distribution of carbonate content. In particular, we observed that carbonate content decreases near the edges of the calcifications closest to neoplastic cells, which may reflect acidified microenvironment as malignant cells proliferate. Using the average carbonate content alone, we achieved a sensitivity of 85% and specificity of 88% in distinguishing benign and neoplastic cases. We expect that the spatial distribution of carbonate content can further improve the sensitivity and specificity in diagnosis. However, in sectioned tissue, the carbonate content pattern is disturbed due to sectioning artifacts (shattering of calcifications and spatial translocation). This problem can be addressed in future studies by imaging either fixed tissue or fresh tissue, where calcifications remain intact.

Besides carbonate content of calcifications, we have elucidated the unique features that distinguish chemical composition of calcifications between FA and IDC. For the first time, we demonstrate that SHG data confirms collagen in FA makes an organic matrix upon which hydroxyapatite accumulates. This matrix observed in FA calcifications is one of the main differences between FA and IDC. This collagen matrix enables robust differentiation of FA and IDC, which exhibit similar carbonate content.

In conclusion, we imaged a diverse set of breast calcifications associated with benign processes, and a range of neoplastic processes including FA, ADH, DCIS and IDC. Our findings support previous reports of carbonate content decreasing on average with increasing malignant potential. Importantly, we showed that a detailed spatial map of calcifications and their complex underlying organic matrix is highly correlated with underlying neoplastic processes and could potentially be used for breast cancer diagnosis. Additionally, the spatial heterogeneity of carbonate content could potentially be a diagnostic indicator of malignancy. Calcification underlying organic matrix, visualized using SHG in conjunction with SRS, assisted in the diagnostic differentiation of patient lesions. Our study suggests that SRS imaging of calcification can be used as an important tool for breast cancer diagnosis. The diagnosis capability will be significantly improved when combined with stimulated Raman histology (SRH), a technique that has already been shown to provide H&E equivalent information in unsectioned tissue 33,35. Importantly, unsectioned tissue, either fixed or fresh, provides comprehensive information on the 3D structure and composition of calcifications, which could provide additional quantitative calcification metrics (size, shape, and chemical distribution) for diagnosis. Coupling calcification and SRH imaging with SHG imaging of collagen matrix, we believe that our comprehensive approach holds tremendous potential in improving the complicated diagnostic process for breast cancer and minimizing inter-pathologist discordance. These advances will ultimately ensure the correct diagnosis and the most effective treatment for the patient.

## Supplementary Material

Supplementary figures and tables.
