# Supplementary material for: Quantitative chemical imaging of breast calcifications in association with neoplastic processes
Source: Theranostics. 2020 Apr 27;10(13):5865–78. doi: 10.7150/thno.43325 (PMC7254998; doi:10.7150/thno.43325)
Supplement: Supplementary file 1 — Supplementary figures and tables. [file thnov10p5865s1.pdf]

Quantitative chemical imaging of breast calcifications in association with neoplastic processes

Kseniya S. Shin[1,2], Mint Laohajaratsang[1], Shuaiqian Men[1], Benjamin Figueroa[1], Suzanne M. Dintzis[3], Dan Fu[1,*]

[1]Department of Chemistry, University of Washington, Seattle, 98195-1700, USA
[2]School of Medicine, University of Washington, Seattle, 98195-1700, USA
[3]Department of Pathology, University of Washington, Seattle, 98195-1700, USA
*danfu@uw.edu

SUPPLEMENTARY MATERIAL

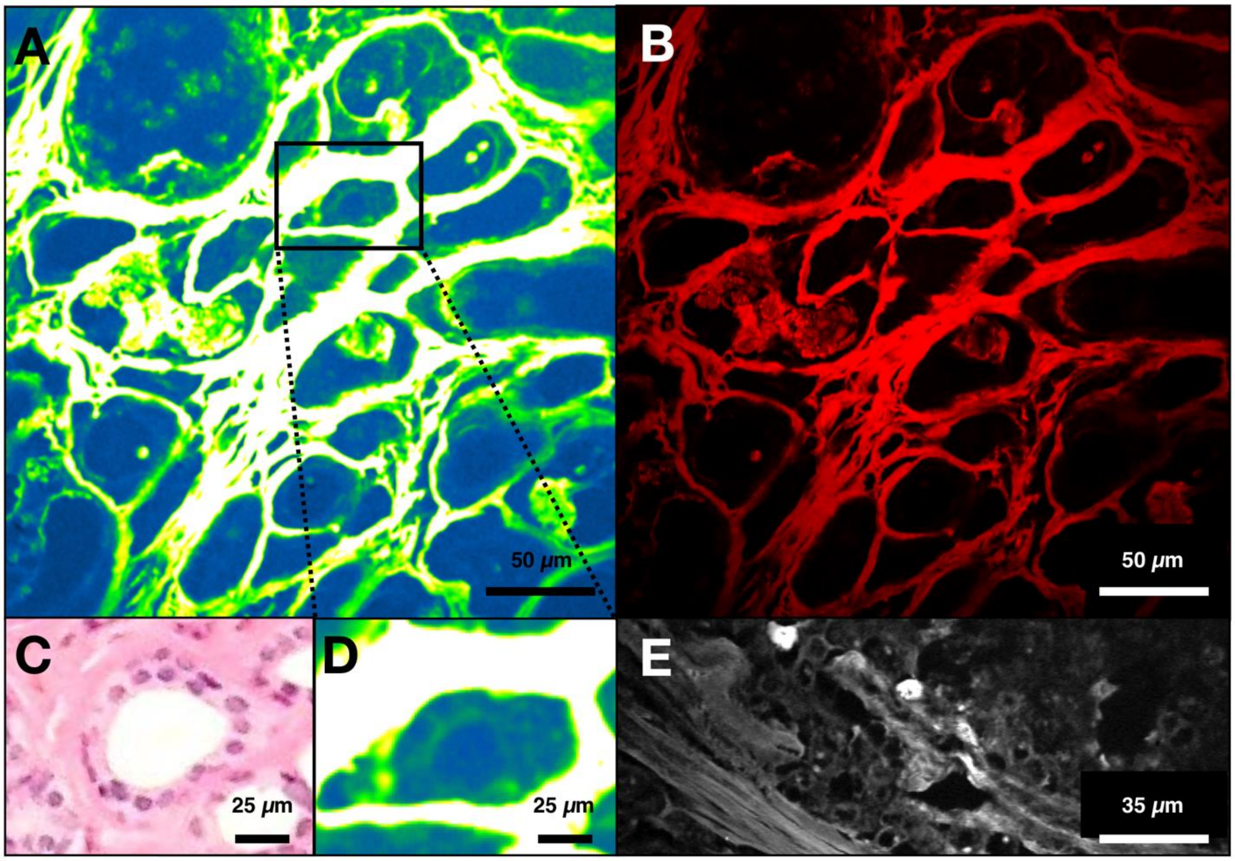

Supplementary Figure 1: Visualizing cellular material in presence of strong CH signal from stromal collagen. A) Adenosis case presented in Figure 3 of main manuscript with adjusted intensity and color scale to assist with visualizing cellular material. B) Adenosis case as presented in Figure 3 of main manuscript. C) A close up of a duct on H&E. D) A close up of a duct from image in A). E) Additional example with easier to visualize cellular material (DCIS case).

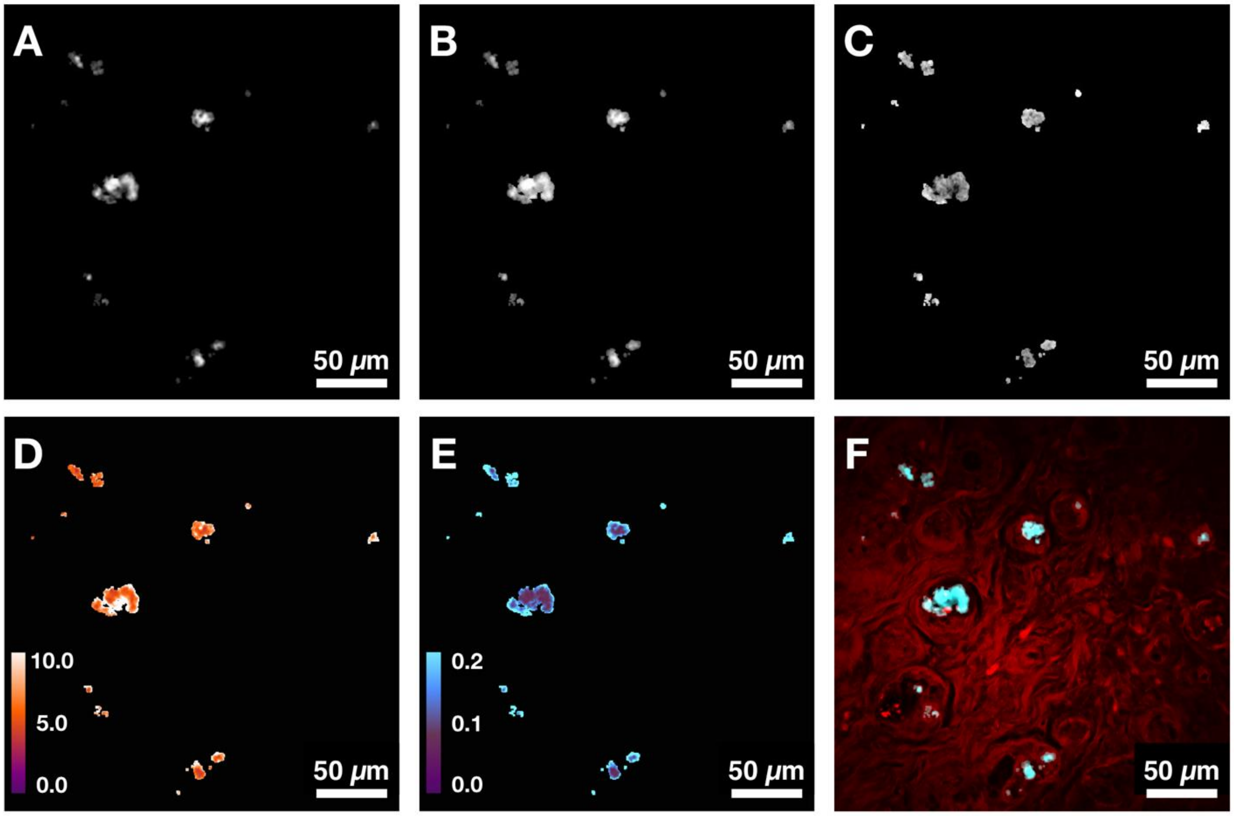

Supplementary Figure 2: Correlating images at phosphate, carbonate, and phenylalanine Raman transitions to resulting carbonate content % and phenylalanine to phosphate ratio maps (calcifications associated with benign process). A) Image at phosphate Raman transition (~960 cm^{-1}). B) Image at carbonate Raman transition (~1070 cm^{-1}). C) Image at phenylalanine Raman transition (~1005 cm^{-1}). D) Carbonate content % map. E) Phenylalanine to phosphate ratio map. F) Image at CH Raman transition (red) together with image at phosphate Raman transition (cyan) to highlight calcification presence in tissue.

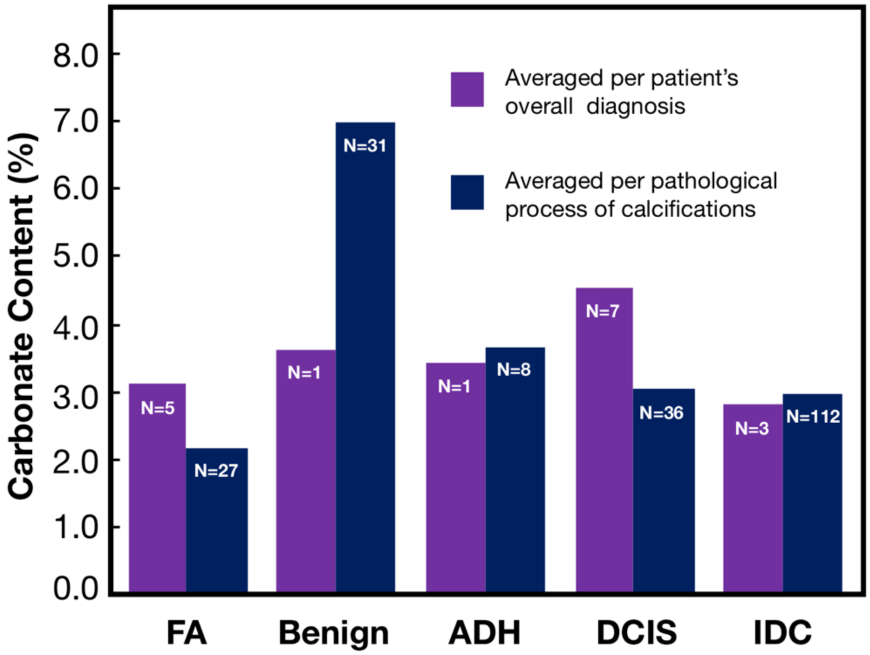

Supplementary Figure 3: Comparing the averages for carbonate content % as calculated per patient's overall diagnosis *versus* per pathological process of calcifications.

Patient	Sex	Age	Specimen type	Overall diagnosis	Number of calcifications associated with specified pathology					Total number of calcifications per patient	Carbonate content % per patient
					Benign	ADH	DCIS	IDC	FA		
1	F	75	Excisional biopsy	FA	0	0	0	0	9	**9**	2.3±1.1
2	F	64	Needle core biopsy	FA	0	0	0	0	11	**11**	2.4±0.8
3	F	49	Needle core biopsy	DCIS	0	0	8	0	0	**8**	4.4±1.8
4	F	56	Needle core biopsy	DCIS	0	0	1	0	0	**1**	3.5
5	F	57	Needle core biopsy	DCIS	0	0	4	0	0	**4**	3.5±0.5
6	F	58	Excision	ADH	0	3	0	0	0	**3**	3.4±0.5
7	F	51	Excision	DCIS	0	3	0	0	0	**3**	3.9±0.3
8	F	60	Needle core biopsy	DCIS	3	0	0	0	0	**3**	7.7±0.3
9	F	40	Needle core biopsy	DCIS	1	2	0	0	0	**3**	5.7±3.9
10	F	59	Needle core biopsy	FA	0	0	0	0	4	**4**	1.6±0.6
11	F	57	Needle core biopsy	FA	18	0	0	0	0	**18**	6.4±1.6
12	F	51	Excision	Adenosis	7	0	0	0	0	**7**	3.6±0.8
13	F	60	Needle core biopsy	DCIS	2	0	19	0	0	**21**	3.8±1.2
14	F	56	Unilateral mastectomy	IDC	0	0	1	76	0	**77**	2.3±0.7
15	F	68	Lumpectomy	IDC	0	0	0	11	0	**11**	3.6±1.1
16	F	56	Unilateral mastectomy	IDC	0	0	3	25	0	**28**	2.5±0.8
17	F	32	Lumpectomy	FA	0	0	0	0	3	**3**	2.9±0.8
**Total number of calcifications per each category**					**31**	**8**	**36**	**112**	**27**		

Supplementary Table 1: A patient clinical history summary together with detailed account of calcification number in each category. Carbonate content % average for each patient is provided.

	Two-sample T-test P-value	
**Categories Compared**	**Carbonate Content %**	**Phenylalanine/Phosphate Ratio**
Benign *versus* DCIS	< 0.001	0.013
Benign *versus* ADH	< 0.001	< 0.001
Benign *versus* IDC	< 0.001	< 0.001
Benign *versus* FA	< 0.001	< 0.001
DCIS *versus* IDC	< 0.001	0.002
ADH *versus* DCIS	0.059	0.021
Benign *versus* all neoplastic	< 0.001	< 0.001
FA *versus* IDC	0.395	0.001

Supplementary Table 2: Summary of t-test p-values for various categories compared (for both carbonate content % and phenylalanine/phosphate ratio). P-value of <0.005 is considered statistically significant and P-value of <0.001 is considered statistically highly significant.
